# Supplementary material for: HIVprotI: an integrated web based platform for prediction and design of HIV proteins inhibitors
Source: J Cheminform. 2018 Mar 9;10:12. doi: 10.1186/s13321-018-0266-y (PMC5845081; doi:10.1186/s13321-018-0266-y)
Supplement: Supplementary file 2 — Additional file 2. Source code of HIVProtI web server. [file 13321_2018_266_MOESM2_ESM.zip › HIVprotI_Source-code/hivprotI/weka-3-6-6/doc/weka/classifiers/mi/SimpleMI.html]

SimpleMI


---


|  |  |  |
| --- | --- | --- |
| **Overview** \| **Package** \| **Class** \| **Tree** \| **Deprecated** \| **Index** \| **Help** \| **Weka's home** | |  |
| **PREV CLASS**   NEXT CLASS | **FRAMES**    **NO FRAMES**     **All Classes** |  |
| SUMMARY: NESTED \| FIELD \| CONSTR \| METHOD | DETAIL: FIELD \| CONSTR \| METHOD |  |


---


## weka.classifiers.mi Class SimpleMI

```
java.lang.Object
  weka.classifiers.Classifier
      weka.classifiers.SingleClassifierEnhancer
          weka.classifiers.mi.SimpleMI
```

**All Implemented Interfaces:**: java.io.Serializable, java.lang.Cloneable, CapabilitiesHandler, MultiInstanceCapabilitiesHandler, OptionHandler, RevisionHandler

---

``` public class SimpleMI extends SingleClassifierEnhancer implements OptionHandler, MultiInstanceCapabilitiesHandler ```

Reduces MI data into mono-instance data.


Valid options are:

```
 -M [1|2|3]
  The method used in transformation:
  1.arithmatic average; 2.geometric centor;
  3.using minimax combined features of a bag (default: 1)
 
  Method 3:
  Define s to be the vector of the coordinate-wise maxima
  and minima of X, ie., 
  s(X)=(minx1, ..., minxm, maxx1, ...,maxxm), transform
  the exemplars into mono-instance which contains attributes
  s(X)
```

```
 -D
  If set, classifier is run in debug mode and
  may output additional info to the console
```

```
 -W
  Full name of base classifier.
  (default: weka.classifiers.rules.ZeroR)
```

```
 Options specific to classifier weka.classifiers.rules.ZeroR:
```

```
 -D
  If set, classifier is run in debug mode and
  may output additional info to the console
```

**Version:**
:   $Revision: 1.6 $

**Author:**
:   Eibe Frank (eibe@cs.waikato.ac.nz), Xin Xu (xx5@cs.waikato.ac.nz), Lin Dong (ld21@cs.waikato.ac.nz)

**See Also:**: Serialized Form

---

| **Field Summary** | |
| --- | --- |
| `static Tag[]` | `TAGS_TRANSFORMMETHOD`             the transformation methods |
| `static int` | `TRANSFORMMETHOD_ARITHMETIC`             arithmetic average |
| `static int` | `TRANSFORMMETHOD_GEOMETRIC`             geometric average |
| `static int` | `TRANSFORMMETHOD_MINIMAX`             using minimax combined features of a bag |


**Constructor Summary**

`SimpleMI()`


| **Method Summary** | |
| --- | --- |
| `void` | `buildClassifier(Instances train)`             Builds the classifier |
| `double[]` | `distributionForInstance(Instance newBag)`             Computes the distribution for a given exemplar |
| `Capabilities` | `getCapabilities()`             Returns default capabilities of the classifier. |
| `Capabilities` | `getMultiInstanceCapabilities()`             Returns the capabilities of this multi-instance classifier for the relational data. |
| `java.lang.String[]` | `getOptions()`             Gets the current settings of the Classifier. |
| `java.lang.String` | `getRevision()`             Returns the revision string. |
| `SelectedTag` | `getTransformMethod()`             Get the method used in transformation. |
| `java.lang.String` | `globalInfo()`             Returns a string describing this filter |
| `java.util.Enumeration` | `listOptions()`             Returns an enumeration describing the available options. |
| `static void` | `main(java.lang.String[] argv)`             Main method for testing this class. |
| `static double[]` | `minimax(Instances data, int attIndex)`             Get the minimal and maximal value of a certain attribute in a certain data |
| `void` | `setOptions(java.lang.String[] options)`             Parses a given list of options. |
| `void` | `setTransformMethod(SelectedTag newMethod)`             Set the method used in transformation. |
| `java.lang.String` | `toString()`             Gets a string describing the classifier. |
| `Instances` | `transform(Instances train)`             Implements MITransform (3 type of transformation) 1.arithmatic average; 2.geometric centor; 3.merge minima and maxima attribute value together |
| `java.lang.String` | `transformMethodTipText()`             Returns the tip text for this property |

**Methods inherited from class weka.classifiers.SingleClassifierEnhancer**

`classifierTipText, getClassifier, setClassifier`

**Methods inherited from class weka.classifiers.Classifier**

`classifyInstance, debugTipText, forName, getDebug, makeCopies, makeCopy, setDebug`

**Methods inherited from class java.lang.Object**

`equals, getClass, hashCode, notify, notifyAll, wait, wait, wait`

**Field Detail**

### TRANSFORMMETHOD\_ARITHMETIC

```
public static final int TRANSFORMMETHOD_ARITHMETIC
```

:   arithmetic average

    **See Also:**: Constant Field Values

---


### TRANSFORMMETHOD\_GEOMETRIC

```
public static final int TRANSFORMMETHOD_GEOMETRIC
```

:   geometric average

    **See Also:**: Constant Field Values

---


### TRANSFORMMETHOD\_MINIMAX

```
public static final int TRANSFORMMETHOD_MINIMAX
```

:   using minimax combined features of a bag

    **See Also:**: Constant Field Values

---


### TAGS\_TRANSFORMMETHOD

```
public static final Tag[] TAGS_TRANSFORMMETHOD
```

:   the transformation methods


**Constructor Detail**

### SimpleMI

```
public SimpleMI()
```


**Method Detail**

### globalInfo

```
public java.lang.String globalInfo()
```

:   Returns a string describing this filter

    :   **Returns:**: a description of the filter suitable for displaying in the explorer/experimenter gui

---


### listOptions

```
public java.util.Enumeration listOptions()
```

:   Returns an enumeration describing the available options.

    :   **Specified by:**: `listOptions` in interface `OptionHandler` **Overrides:**: `listOptions` in class `SingleClassifierEnhancer`
    :   **Returns:**: an enumeration of all the available options.

---


### setOptions

```
public void setOptions(java.lang.String[] options)
                throws java.lang.Exception
```

:   Parses a given list of options.


    Valid options are:

    ```
     -M [1|2|3]
      The method used in transformation:
      1.arithmatic average; 2.geometric centor;
      3.using minimax combined features of a bag (default: 1)
     
      Method 3:
      Define s to be the vector of the coordinate-wise maxima
      and minima of X, ie., 
      s(X)=(minx1, ..., minxm, maxx1, ...,maxxm), transform
      the exemplars into mono-instance which contains attributes
      s(X)
    ```

    ```
     -D
      If set, classifier is run in debug mode and
      may output additional info to the console
    ```

    ```
     -W
      Full name of base classifier.
      (default: weka.classifiers.rules.ZeroR)
    ```

    ```
     Options specific to classifier weka.classifiers.rules.ZeroR:
    ```

    ```
     -D
      If set, classifier is run in debug mode and
      may output additional info to the console
    ```

    :   **Specified by:**: `setOptions` in interface `OptionHandler` **Overrides:**: `setOptions` in class `SingleClassifierEnhancer`
    :   **Parameters:**: `options` - the list of options as an array of strings **Throws:**: `java.lang.Exception` - if an option is not supported

---


### getOptions

```
public java.lang.String[] getOptions()
```

:   Gets the current settings of the Classifier.

    :   **Specified by:**: `getOptions` in interface `OptionHandler` **Overrides:**: `getOptions` in class `SingleClassifierEnhancer`
    :   **Returns:**: an array of strings suitable for passing to setOptions

---


### transformMethodTipText

```
public java.lang.String transformMethodTipText()
```

:   Returns the tip text for this property

    :   **Returns:**: tip text for this property suitable for displaying in the explorer/experimenter gui

---


### setTransformMethod

```
public void setTransformMethod(SelectedTag newMethod)
```

:   Set the method used in transformation.

    :   **Parameters:**: `newMethod` - the index of method to use.

---


### getTransformMethod

```
public SelectedTag getTransformMethod()
```

:   Get the method used in transformation.

    :   **Returns:**: the index of method used.

---


### transform

```
public Instances transform(Instances train)
                    throws java.lang.Exception
```

:   Implements MITransform (3 type of transformation) 1.arithmatic average;
    2.geometric centor; 3.merge minima and maxima attribute value together

    :   **Parameters:**: `train` - the multi-instance dataset (with relational attribute) **Returns:**: the transformed dataset with each bag contain mono-instance (without relational attribute) so that any classifier not for MI dataset can be applied on it. **Throws:**: `java.lang.Exception` - if the transformation fails

---


### minimax

```
public static double[] minimax(Instances data,
                               int attIndex)
```

:   Get the minimal and maximal value of a certain attribute in a certain data

    :   **Parameters:**: `data` - the data: `attIndex` - the index of the attribute **Returns:**: the double array containing in entry 0 for min and 1 for max.

---


### getCapabilities

```
public Capabilities getCapabilities()
```

:   Returns default capabilities of the classifier.

    :   **Specified by:**: `getCapabilities` in interface `CapabilitiesHandler` **Overrides:**: `getCapabilities` in class `SingleClassifierEnhancer`
    :   **Returns:**: the capabilities of this classifier **See Also:**: `Capabilities`

---


### getMultiInstanceCapabilities

```
public Capabilities getMultiInstanceCapabilities()
```

:   Returns the capabilities of this multi-instance classifier for the
    relational data.

    :   **Specified by:**: `getMultiInstanceCapabilities` in interface `MultiInstanceCapabilitiesHandler`
    :   **Returns:**: the capabilities of this object **See Also:**: `Capabilities`

---


### buildClassifier

```
public void buildClassifier(Instances train)
                     throws java.lang.Exception
```

:   Builds the classifier

    :   **Specified by:**: `buildClassifier` in class `Classifier`
    :   **Parameters:**: `train` - the training data to be used for generating the boosted classifier. **Throws:**: `java.lang.Exception` - if the classifier could not be built successfully

---


### distributionForInstance

```
public double[] distributionForInstance(Instance newBag)
                                 throws java.lang.Exception
```

:   Computes the distribution for a given exemplar

    :   **Overrides:**: `distributionForInstance` in class `Classifier`
    :   **Parameters:**: `newBag` - the exemplar for which distribution is computed **Returns:**: the distribution **Throws:**: `java.lang.Exception` - if the distribution can't be computed successfully

---


### toString

```
public java.lang.String toString()
```

:   Gets a string describing the classifier.

    :   **Overrides:**: `toString` in class `java.lang.Object`
    :   **Returns:**: a string describing the classifer built.

---


### getRevision

```
public java.lang.String getRevision()
```

:   Returns the revision string.

    :   **Specified by:**: `getRevision` in interface `RevisionHandler` **Overrides:**: `getRevision` in class `Classifier`
    :   **Returns:**: the revision

---


### main

```
public static void main(java.lang.String[] argv)
```

:   Main method for testing this class.

    :   **Parameters:**: `argv` - should contain the command line arguments to the scheme (see Evaluation)


---


|  |  |  |
| --- | --- | --- |
| **Overview** \| **Package** \| **Class** \| **Tree** \| **Deprecated** \| **Index** \| **Help** \| **Weka's home** | |  |
| **PREV CLASS**   NEXT CLASS | **FRAMES**    **NO FRAMES**     **All Classes** |  |
| SUMMARY: NESTED \| FIELD \| CONSTR \| METHOD | DETAIL: FIELD \| CONSTR \| METHOD |  |


---
